# Supplementary material for: Signal mining and analysis of adverse events of Brentuximab Vedotin base on FAERS and JADER databases
Source: PLoS One. 2025 May 16;20(5):e0322378. doi: 10.1371/journal.pone.0322378 (PMC12083808; doi:10.1371/journal.pone.0322378)
Supplement: Attachment — Submitted filename: Response to Reviewers.docx [file pone.0322378.s003.docx]

Dear reviewers and editors,

Thank you for your letter and for the reviewers’ comments concerning our manuscript entitled “Signal mining and analysis of adverse events of Brentuximab Vedotin base on FAERS and JADER database” (ID: PONE-D-24-42713). Those comments are all valuable and very helpful for revising and improving our paper, as well as the important guiding significance to our researches. We have studied comments carefully and have made corrections which we hope to meet with approval. Revised portions are marked in red on the paper. The corrections in the paper and the responses to the reviewer’s comments are as flowing:

**To reviewer #1:**

**Comment 1:** In this manuscript, the authors analysed safety data from 2 AE data bases, namely the FAERS (USA) and the JADER (Japan) in relation to an approved therapeutic agent for haematologic malignancies, the mAb-drug conjugate Brentuximab Vedotin (BV).

The authors followed a standard methodology for data mining and analysis.

The results are supported by the corresponding Tables and Figures.

In the discussion section, the findings are described in a comprehensive way, where the authors also attempt to explain the results as well as the discrepancies and casual relationship.

Despite the study limitations, as clearly described and admitted in section 4.5., the paper provides convincing and clinically useful data as to the safety profile of BV. It is important that the information is derived from both clinical trials and real world (postmarketing) data.

**Response 1:** Thank you for your careful review and approval.

**To reviewer #2:**

**Comment 1:** In the current manuscript authors studied adverse events related to Brentuximab Vedotin treatment in patients. The manuscript provides broad analysis of observed adverse events for different physiological systems reported in clinical data reported by FAERS & JADER. It is expected to observe adverse events with ADCs for Blood and Lymphatics system disorders while observed findings suggests that BV Has minimal ROR for Nervous system disorders in FEARS reported studies while it has significantly high ROR in JADER reported study. Is this discrepancy due to demography factor or the state of disease and administered dose has some role in this?

**Response 1:** Thanks for your careful review and suggestion. The results of BV monotherapy for relapsed or refractory Hodgkin lymphoma: a systematic review and meta-analysis (PMID: 34323643) indicated that the most common AEs were hematological toxicities (neutropenia: 13.3-23%, anemia: 8.8-39.0%, and thrombocytopenia: 4-4.6%), and grade ≥3 peripheral neuropathy. In this study, hematological toxicity was reflected in both SOC and PT, and the frequency and intensity were high, which was consistent with the literature reports. Neurological AEs occurred with greater intensity in the two databases, with lower intensity in the FAERS database (n = 1476, ROR = 1.07) and higher intensity in the JADER database (n = 481, ROR = 2.39) (Fig 1). Studies have shown that: Each component of the ADC, including the antibody, linker, and payload, may affect the extent of the ADC-induced toxicities (PMID: 36765668). We reviewed a large number of studies on BV-induced neurotoxicity and found that there are few reports on the analysis of factors causing differences in neurotoxicity. A study suggests that BV-induced neurotoxicity (BVIN) is a known cumulative adverse event, The level and duration of MMAE exposure in the peripheral nerve tissue is believed to be the most important determinant of PN (Peripheral Neurotoxicity). Risk factors for BVINs may include individual patient factors including demographics and patient characteristics, pre-existing conditions or prior anti-cancer therapies, concurrent medications or nutritional supplements (i.e., pyridoxine) or herbal preparations (PMID: [34885234](https://pubmed.ncbi.nlm.nih.gov/34885234/)). In view of the limited information available, we add to the discussion [It is notable that BV has minimal ROR for nervous system disorders in FAERS reported studies while it has significantly high ROR in JADER reported study. Further studies are required to ascertain whether the reasons for this discrepancy are associated with demographic factors, disease state, and dose administered.] (page 17, line 302-306).

**Comment 2:** Authors provided a method to calculate ROR while authors should mention if they have used any specific software to perform these calculations.

**Response 2:** Thanks for the suggestion. The calculations were performed by editing the ROR formula using Excel 2019. A detailed description of what analyses were performed using Excel has been provided (page 6, line 162). Function editing is shown in the figure below:

**Comment 3:** Authors mentioned using R and Excel for performing data analysis, I think author should elaborate which analysis was performed by excel and R. Further mention any R Package used for analysis if applicable.

**Response 3:** Thank you for this comment. We’ve changed [Data processing and statistical analysis were performed using Microsoft Excel 2019 and R 4.4.2.] to [All data extraction, de-duplication, cleaning, and graphing were conducted using R 4.2.2 software, while the mapping, intensity calculations, and sorting of the data were performed using Microsoft Excel 2019 software.] (page 6, line 161-164).

**To reviewer #3:**

**Major comments:**

**Comment 1:** Data Source: The rationale of selecting “period from Q1 2004 to Q1 2023” remains unclear. In other words, c and d reflect a time period different from that when the Brentuximab Vedotin was utilized which introduces bias.

**Response 1:** Thanks for your careful review and suggestion. We agree with this comment of the reviewers: the inconsistency of the time ranges extracted from the two databases does introduce bias. However, we believe that updating the FAERS database is neither feasible nor would it have a significant impact on our argument, given the costs involved. Moreover, we reviewed the literature on mining AEs using two databases and found that in some articles the time ranges extracted from the two databases were also inconsistent (e.g., PMID: 39144633, PMID: 31595301, PMID: 39635439). For this reason, we chose not to make this change, for which we apologize. We will take this into consideration when we conduct similar studies in the future, and try to adjust the time ranges as much as possible to minimize the bias, thanks again.

**Comment 2:** Kindly add to table 1. “Indications for use or diagnosis (INDI)” i.e., how the frequency of AEs varied in Relapsed versus previously untreated. Also, the frequency of AEs in different diagnoses.

**Response 2:** Thank you for this comment. The FAERS and JADER databases cover too many Indications for use or diagnosis (INDI), 161 and 712, respectively. We classified and excluded descriptions unrelated to the INDI, such as Product used for unknown indication, Not Specified, Illness, etc. The top 5 frequencies for INDI were listed and added to table 1.

**Comment 3:** Signals of preferred terms; you mention that you excluded “signals unrelated to adverse drug reactions, such as injuries, poisoning, procedural complications, surgical and medical procedures, social circumstances, and product issues” however, Figure 3B still shows “femoral neck fracture” with SOC “Injury, poisoning and procedural complications”. In the case that specific PTs were excluded and not every PT under the mentioned SOC, please make it more clear in the text.

**Response 3:** Thanks for your careful review and suggestion. We rechecked the data and found that the “Injury, poisoning and procedural complications” system was not fully excluded, resulting in “femoral neck fracture” appeared in the figure 3B. We apologize for this error. We have corrected the error and redrawn the figure 3.

**Comment 4:** Duplicate reporting is mentioned as a limitation. It was mentioned earlier in the manuscript that “de-duplication method”. Kindly elaborate.

**Response 4:** Thanks for the suggestion. The officially recommended method of de-duplication has been described in detail, specifically [Specifically, for reports with the same CASEID, the report with the largest FDA_DT value was retained, and for reports with the same CASEID and FDA_DT, the report with the largest PRIMARYID value was selected.] (page 5, line 129-132).

**Comment 5:** Additional analysis recommended as feasible for the following:

• No statistical tests were carried to measure the agreement between data generated from FARES and JARES. Consider conducting comparative analysis for shared AEs and for PTs with positive signals.

• For the unexpected AEs, a detailed analysis of the patient population “Indications for use or diagnosis (INDI)” would provide clinical meaningfulness.

• Onset time of BV-related AEs. Kindly elaborate on the time of onset of the PTs with positive signals. Additionally, please add details on the type of AE observed at or after 1 year.

**Response 5:** Thank you for the valuable suggestion. We agree with the reviewers that adding the analysis mentioned above would give the manuscript more depth. Firstly, thank you to the reviewer for prompting us to measure the consistency between the data generated by FARES and JADER through statistical tests. Our team was not previously aware of this idea, but through a literature review, we found a paper that used the chi-square test to assess whether there was a significant correlation between data from the two databases [PMID: 39402995]. We apologize that we were unable to assess the consistency of the data this time due to the difficulty of the computational operation. Secondly, the two databases involve a large number of unexpected AEs and Indications for use or diagnosis (INDI), and the relationship is complex, so it is difficult to achieve visualization and detailed analysis. Finally, the occurrence time of AEs is seriously missing in both databases, especially in the JADER database. The positive signals in the two databases are different in type and quantity, and the analysis of the occurrence time of the positive signals in the two databases has not been reported, so it is difficult to operate. In the future study, we will perform related studies for deeply and thoroughly understand those valuable suggestion. Thank you very much for this creative idea again.

**Further comments:**

**Introduction**

**Comment 6:** “On August 19, 2011….. systemic anaplastic large cell lymphoma (sALCL)”. It is recommended to make it clear that the initial approval was for relapsed refractory while that for previously untreated was recently granted in 2018. Since you referenced cutaneous T-cell Lymphoma in the discussion, you might consider including it in the indications mentioned.

**Response 6:** We are grateful for the comment. A meticulous examination of the drug's FDA marketing history and approved indications has been conducted, and the paragraph description has been revised in accordance with the necessary amendments.

We’ve changed [On August 19, 2011, the U.S. Food and Drug Administration (FDA) granted BV its first approval for the treatment of classical Hodgkin lymphoma (cHL) and systemic anaplastic large cell lymphoma (sALCL).] to [On August 19, 2011, the U.S. Food and Drug Administration (FDA) granted BV its first approval for the treatment of relapsed Hodgkin lymphoma (HL) and systemic anaplastic large cell lymphoma (sALCL). In 2018, the drug was extended with new indications for the treatment of Previously untreated classical Hodgkin lymphoma (cHL) and CD30-expressing peripheral T-cell lymphomas (PTCL).] (page 3, line 78-83).

**Comment 7:** In page 4 “severe “ insulin resistance [9] …..ketoacidosis [12]. Please clarify that the data was obtained from case-studies. It would further support the rationale for conducting the study.

**Response 7:** We are grateful for the suggestion. We have changed [Most of these AEs are reversible and manageable; however, rare but potentially life-threatening AEs have been reported, such as …..] to [Most of these AEs are reversible and manageable; however, rare but potentially life-threatening AEs have been reported from case-studies, such as…..] (page 4, line 94-98).

**Results and Discussion**

**Comment 8:** “In terms of age, the FAERS database was predominantly composed of individuals aged 18–45 (1,159, 19.82%), whereas the JADER database was mainly represented by individuals aged ≥65 (811, 43.18%)”. The age data appears to be missing in almost 50% of cases in the FARES database, making it hard to conclude that Brentuximab Vedotin is more predominant in 18-45 yrs age group. Kindly rephrase to make this clear or elsewise remove the comment on the FARES database.

This also applies to “In terms of age distribution, the FAERS database showed the highest proportion of patients aged 18–45 years, aligning with the well-established prevalence of HL in young adults” in the discussion section.

**Response 8:** Thanks for the reviewer careful review and suggestion. The age distribution is re-described in the results and analysed accordingly in the discussion. New references changed see [20] Ansell SM. Hodgkin lymphoma: 2025 update on diagnosis, risk-stratification, and management. Am J Hematol. 2024; 99(12):2367-2378. <https://doi.org/10.1002/ajh.27470> PMID: 39239794. We have changed [In terms of age, the FAERS database was predominantly composed of individuals aged 18–45 (1,159, 19.82%), whereas the JADER database was mainly represented by individuals aged ≥65 (811, 43.18%).] to [In terms of age, the FAERS database had significant missing age data (2860, 48.90%). Excluding missing data, the FAERS database was predominantly composed of individuals aged 18–45 (1,159, 19.82%), whereas the JADER database was mainly represented by individuals aged ≥65 (811, 43.18%).] (page 7, line 180-183) in results, and we have changed [In terms of age distribution, the FAERS database showed the highest proportion of patients aged 18–45 years, aligning with the well-established prevalence of HL in young adults [20]. In contrast, the JADER database had a higher proportion of patients aged ≥65 years, a finding that warrants further epidemiological investigation.] to [The FAERS and JADER databases indicate a predominant age concentration of 18-45 years and >65 years, respectively, which is consistent with a bimodal distribution of HL, with a higher incidence in younger people and in patients aged 55 years and older [20]. In light of the potential impact of missing data on the findings of this study, further research is required to ascertain whether AEs are age-related.] (page 17, line 302-306) in discussion.

**Comment 9:** "Concerning serious clinical outcomes, the FAERS database had the highest percentage of hospitalization cases (2,477, 42.35%), followed by deaths (1,368, 23.39%). In contrast, the JADER database demonstrated a definitive outcome of death (429, 22.84%)" relays that the two databases were compared. Please consider rephrasing.

**Response 9:** We apologize for the failure to accurately describe the serious clinical outcomes in the manuscripts. The occurrence of serious clinical outcomes in two databases has been redescribed. We have changed [Concerning serious clinical outcomes, the FAERS database had the highest percentage of hospitalization cases (2,477, 42.35%), followed by deaths (1,368, 23.39%). In contrast, the JADER database demonstrated a definitive outcome of death (429, 22.84%).] to [Concerning serious clinical outcomes, the FAERS database had the highest percentage of hospitalization cases (2,477, 42.35%), followed by death (1,368, 23.39%). In contrast, the JADER database only provided clinical results for death cases (429, 22.84%), with no information available for other outcomes.] (page 7-8, line 188-193).

**Comment 10:** Figure 2A & 2B; x-axis kindly modify "number" to “number of events”.

**Response 10:** Thanks for your careful review. We have revised it.

**Comment 11:** For the sake of clarity, it is recommended to shuffle column 1 & 2 in figure 3A & 3B i.e., PT as the first column. Please clarify what star and triangle signify in the figures.

**Response 11:** Thanks for the suggestion. We have re-drawn Fig 3A & 3B as required. An explanation of the meaning of stars and triangles in the diagram has been added.

**Comment 12:** Consider removing the detailed monitoring/dose modification recommendation from the discussion section. You might consider adding general recommendation in the conclusion.

**Response 12:** Thanks for the reviewer careful review and suggestion. We removed some of the detailed monitoring from the discussion, specifically [Patients experiencing any ocular discomfort or changes in vision during treatment should seek immediate medical attention. Physicians must weigh the benefits and risks of treatment based on the patient's condition and adjust the treatment regimen as needed] (page 22, line 424-427) and [Although the number of reported cases is relatively small, the severity of these events is notable, and further investigation is needed to determine whether a true causal relationship exists. Clinicians must remain vigilant in conducting timely examinations and taking appropriate therapeutic measures to prevent the occurrence of serious AEs] (page 22, line 439-443). General recommendations have been added to the conclusion section, specifically [When AEs are suspected, close attention should be paid and a timely diagnosis made. If AEs are identified, the appropriate management plan should be initiated, such as adjustment of the drug regimen, postponement of treatment or dose reduction, and timely discontinuation of the drug. In the event of an untreatable AE, a multidisciplinary consultation should be arranged immediately to explore solutions.] (page 23-24, line 479-483).

**Comment 13:** “Several significant AE signals associated with Brentuximab Vedotin were identified, including death, febrile bone marrow aplasia, Guillain-Barré syndrome, retinal detachment, and femoral neck fracture.” is repeated across the manuscript. Consider rephrasing. You might choose to remove the detailed phrase from the conclusion and keep general recommendations.

**Response 13:** Thanks for the suggestion. We have changed [Several significant AE signals associated with BV were identified, including death, febrile bone marrow aplasia, Guillain-Barré syndrome, and retinal detachment. These findings highlight the importance of maintaining vigilance for potential adverse reactions, particularly during the early stages of treatment.] to [A number of noteworthy AE signals associated with BV have been identified, which serve to underscore the necessity of maintaining a high level of vigilance for potential adverse reactions, particularly during the initial stages of treatment.] (page 23-24, line 472-479).

**To associate Editors:**

**Comment 1:** These adverse effects are related to MMAE; therefore, these findings may be compared to the adverse effects of other MMAE-containing ADCs or may help manage or prevent their side effects. (for example polatuzumab vedotin)

**Response 1:** Thank you for the valuable suggestion. This is a good idea and as a next step we can use several databases (FAERS, VigiAccess, JADER) to compare the differences in MMAE-containing ADCs (brentuximab vedotin, polatuzumab vedotin, enfortumab vedotin, tisotumab vedotin, disitamab vedotin) differences in associated AEs or may help manage or prevent their side effects. The results of a study comparing several MMAE-based ADCs related to Peripheral neuropathy are added to the discussion and described in detail as [Studies have shown that the incidence of any grade of peripheral neuropathy in patients treated with BV ranged from 36% to 69%, with severe peripheral neuropathy occurring in approximately 4-14% of patients. A similar frequency was seen in patients treated with enfortumab vedotin. While polatuzumab vedotin can also cause predominantly sensory peripheral neuropathy, which is typically mild.] (page 18, line 320-323).

**Comment 2:** The discussion part is a little bit repeat of the results. It would be more appropriate if the discussion were better organized and discussed based on current data.

**Response 2:** Thanks for the suggestion. It is true that we put too much emphasis on the results in the discussion section, which led to appearing too repetitive. We have reworded it in the discussion and trimmed it accordingly. What we have deleted are [In this study, the AEs with the highest signal intensity for neurological disorders were peripheral motor neuropathy, peripheral sensory neuropathy, peripheral neuropathy, and polyneuropathy. Given the prevalence and impact of these neurological AEs] (page 18, line 328-331), [signals of haematotoxicity were detected for febrile neutropenia, neutropenia, pancytopenia, and thrombocytopenia] (page 19, line 342-343), and [In such cases, the frequency of testing should be increased.] (page 19, line 349-350). We are also adding [The temporal relationship between drug administration and time to onset is of paramount importance in the assessment of drug safety. A deeper comprehension of the mechanism underlying AEs is vital, as it could facilitate the identification of specific patient groups at risk and specific risk windows in the course of treatment, and contribute to the prevention or earlier diagnosis of AEs] (page 18, line 445-449) to the discussion adding literature 36.

**Comment 3:** For the conclusion part, what do you suggest to help manage these adverse effects and to better report and group them? I think this topic is lacking.

**Response 3:** Thanks for the suggestion. We do lack the topic of the management aspect of AEs in the conclusion. We have added relevant content to the conclusions, specifically [When AEs are suspected, close attention should be paid and a timely diagnosis made. If AEs are identified, the appropriate management plan should be initiated, such as adjustment of the drug regimen, postponement of treatment or dose reduction, and timely discontinuation of the drug. In the event of an untreatable AE, a multidisciplinary consultation should be arranged immediately to explore solutions.] (page 23-24, line 479-483).

**Comment 4:** Please state what role the funders took in the study.  If the funders had no role, please state: "The funders had no role in study design, data collection and analysis, decision to publish, or preparation of the manuscript."

**Response 4:** Thanks for the suggestion. We add the clarification that [The funders had no role in study design, data collection and analysis, decision to publish, or preparation of the manuscript.] (page 29, line 657-659).

Thank you very much for your attention and time. Look forward to hearing from you.

Yours sincerely,

Hongying Xia

Dec 9th, 2024

Department of Pharmacy, Yan'an Hospital Affiliated to Kunming Medical University, Kunming, China.

Tel: 0871-63211154

E-mail: [398872956@qq.com](mailto:398872956@qq.com)
