# Supplementary material for: Signal mining and analysis of adverse events of Brentuximab Vedotin base on FAERS and JADER databases
Source: PLoS One. 2025 May 16;20(5):e0322378. doi: 10.1371/journal.pone.0322378 (PMC12083808; doi:10.1371/journal.pone.0322378)
Supplement: Attachment — Submitted filename: Response to Reviewers.doc [file pone.0322378.s004.doc]

Dear reviewers and editors,

Thank you for your letter and for the reviewers’ comments concerning our manuscript entitled “Signal mining and analysis of adverse events of Brentuximab Vedotin base on FAERS and JADER database” (ID: PONE-D-24-42713). Those comments are all valuable and very helpful for revising and improving our paper, as well as the important guiding significance to our researches. We have studied comments carefully and have made corrections which we hope to meet with approval. Revised portions are marked in red on the paper. The corrections in the paper and the responses to the reviewer’s comments are as flowing:

**To reviewer #1:**

**Comment 1:** Going through the authors' response to the reviewers' comments, l believe that these had been taken into the account and the manuscript is significantly improved.

**Response 1:** Thank you for your careful review and approval.

**To reviewer #3:**

**Major comments:**

**Comment 1:** I am herein clarifying this major comment and request that the authors kindly address it. As mentioned in the manuscript, Brentuximab Vedotin was first approved in 2011. lt remains unclear why the analysis included the time period from 2004 to 2011. Your analysis is based on a relationship between AEs reported for Brentuximab Vedotin compared to that for other drugs during the same time period. lt is expected that there were no AEs reported for Brentuximab Vedotin before 2011; hence bias is introduced into the equation. I believe the ROR calculation will change if events were considered only starting the introduction of Brentuximab Vedotin i.e., starting 2011.

The difference between FARES and JADER i.e., Q1 vs Q4 2023 is not a major concern.

**Response 1:** Thanks for clarifying the comment again. We agree that it would be more reasonable to set the starting point for data retrieval at the quarter in which the month in which the drug was launched. However, due to the technical limitations of our data mining, we were unable to change the time frame for data retrieval from the JADER database, and therefore could only retrieve data from the time of database construction (Q1 2004) to the most recent data at the time of mining (Q4 2023). In order to try to keep the excavated data as consistent as possible, we also pulled data from the FAERS database from 2004 to 2011. As questioned by the reviewers, this may have expanded the number of background drugs, which in turn led to changes in the calculation of ROR. We asked the relevant statisticians and they agreed that the calculation of the ROR would change but would have little impact on the overall trend of the conclusions. We also reviewed a large amount of related literature and found that in some articles the drugs were marketed after 2004, but the starting point of their data mining was set to Q1 2004, such as sorafenib (first marketed in 2005, PMID: 39144633), eltrombopag (first marketed in 2008, PMID: 39144633), tremelimumab (first marketed in 2022, PMID: 39144633), durvalumab (earliest marketed 2017, PMID: 39696525), and upadacitinib (earliest marketed 2019, PMID: 37663269). We will take this into account when conducting similar studies in the future, optimize our data mining techniques, and try to adjust the timeframe to minimize bias, thanks again.

**Comment 4:** Thanks for clarifying the de-duplication method. The question remains, if you applied de-duplication, why is duplicate reporting a limitation?

**Response 4:** Thank you for the valuable suggestion. We did utilize the official FDA recommended de-duplication method for de-duplication and duplicate reports are no longer a limitation. We’ve changed [The data may not be comprehensive, and the quality of reporting varied, with potential biases such as duplicate reporting and underreporting] to [The data may not be comprehensive, and the quality of reporting varied, with potential biases such as underreporting] (page 21, line 438-439).

**Comment 5:** Thank you for your response. It is quite understandable that further analysis might not be feasible. I appreciate that you add your response to the discussion section as a limitation and/or future considerations.

**Response 5:** Thank you for your understanding and satisfaction with our response to this comment. We’ve changed [The time to onset analysis did not capture all cases, as reports with incomplete dates were excluded] to [The time to onset analysis did not capture all cases, as reports with incomplete dates were excluded, especially in the JADER database] (page 21, line 442-444). We are also adding [(6) No statistical tests were carried out to measure the agreement between data generated from FARES and JADER; (7) The two databases involve a large number of unexpected AEs and INDI, and the relationship is complex, so it is difficult to achieve visualization and detailed analysis; (8) The positive signals exhibited variability in terms of their type and quantity across the two databases, thereby impeding the analysis of their temporal occurrence. In future studies, the time of onset of positively signaled PTs and the type of AEs observed beyond one year will be further analyzed. The chi-square test will be used for evaluation to assess whether there was a significant correlation between data from the two databases.] (page 21-22, line 445-454) to the discussion adding literature 39 [PMID: 39402995].

**To associate Editors:**

**Comment 1:** The authors should correct Indication of the reason for the time period chosen for the study and reporting de-duplication as a limitation and also should get an input from a statistician for the ROR analysis covering time period prior drug approval.

**Response 1:** Thank you for the valuable suggestion. As reviewer 3 asked the question, we agree that it would be more reasonable to set the starting point for data retrieval at the quarter in which the month in which the drug was launched. However, due to the technical limitations of our data mining, we were unable to change the time frame for data retrieval from the JADER database, and therefore could only retrieve data from the time of database construction (Q1 2004) to the most recent data at the time of mining (Q4 2023). In order to try to keep the excavated data as consistent as possible, we also pulled data from the FAERS database from 2004 to 2011. As questioned by the reviewers, this may have expanded the number of background drugs, which in turn led to changes in the calculation of ROR. We asked the relevant statisticians and they agreed that the calculation of the ROR would change but would have little impact on the overall trend of the conclusions. We also reviewed a large amount of related literature and found that in some articles the drugs were marketed after 2004, but the starting point of their data mining was set to Q1 2004, such as sorafenib (first marketed in 2005, PMID: 39144633), eltrombopag (first marketed in 2008, PMID: 39144633), tremelimumab (first marketed in 2022, PMID: 39144633), durvalumab (earliest marketed 2017, PMID: 39696525), and upadacitinib (earliest marketed 2019, PMID: 37663269). We will take this into account when conducting similar studies in the future, optimize our data mining techniques, and try to adjust the timeframe to minimize bias.

We did utilize the official FDA recommended de-duplication method for de-duplication and duplicate reports are no longer a limitation. We’ve changed [The data may not be comprehensive, and the quality of reporting varied, with potential biases such as duplicate reporting and underreporting] to [The data may not be comprehensive, and the quality of reporting varied, with potential biases such as underreporting] (page 21, line 438-439).

Thank you very much for your attention and time. Look forward to hearing from you.

Yours sincerely,

Hongying Xia

Feb 17th, 2025

Department of Pharmacy, Yan'an Hospital Affiliated to Kunming Medical University, Kunming, China.

Tel: 0871-63211154

E-mail: [398872956@qq.com](mailto:398872956@qq.com)
