# Supplementary material for: Signal mining and analysis of adverse events of Brentuximab Vedotin base on FAERS and JADER databases
Source: PLoS One. 2025 May 16;20(5):e0322378. doi: 10.1371/journal.pone.0322378 (PMC12083808; doi:10.1371/journal.pone.0322378)
Supplement: Attachment — Submitted filename: Response_to_Reviewers_auresp_3.docx [file pone.0322378.s005.docx]

Dear reviewers and editors,

Thank you for your letter and for the reviewers’ comments concerning our manuscript entitled “Signal mining and analysis of adverse events of Brentuximab Vedotin base on FAERS and JADER database” (ID: PONE-D-24-42713). Those comments are all valuable and very helpful for revising and improving our paper, as well as the important guiding significance to our researches. We have studied comments carefully and have made corrections which we hope to meet with approval. Revised portions are marked in red on the paper. The corrections in the paper and the responses to the reviewer’s comments are as flowing:

**To reviewer #3:** Thank you for your response to comment #1. Please add your explanation to the limitations section.

Here's my suggestion "Due to the technical limitations of our data mining technique, we could only retrieve data from the time of database construction (Q1 2004) to the most recent data at the time of mining (Q4 2023). The analysis a time period prior to the approval of Brentuximab Vedotin, consequently expanding the number of background drugs and leading to changes in the calculation of ROR. However, we believe this had only a little impact on the overall trend of the conclusions."

**Response:** Thank you for your valuable suggestion and satisfaction with our response to this comment. We have added [(9) Due to the technical limitations of our data mining technique, we could only retrieve data from the time of database construction (Q1 2004) to the most recent data at the time of mining (Q4 2023). The analysis a time period prior to the approval of BV, consequently expanding the number of background drugs and leading to changes in the calculation of ROR. However, we believe this had only a little impact on the overall trend of the conclusions.] (page 22, line 451-456).

**To associate Editors:**

**Comment:** All quotes have been addressed however, kindly include your justification in the section on limitations as the reviewer requested.

**Response:** Thanks for the reviewer careful review and suggestion. We have added [(9) Due to the technical limitations of our data mining technique, we could only retrieve data from the time of database construction (Q1 2004) to the most recent data at the time of mining (Q4 2023). The analysis a time period prior to the approval of BV, consequently expanding the number of background drugs and leading to changes in the calculation of ROR. However, we believe this had only a little impact on the overall trend of the conclusions.] (page 22, line 451-456) to the limitations section of the manuscript as suggested by reviewer #3.

Thank you very much for your attention and time. Look forward to hearing from you.

Yours sincerely,

Hongying Xia

Mar 18th, 2025

Department of Pharmacy, Yan'an Hospital Affiliated to Kunming Medical University, Kunming, China.

Tel: 0871-63211154

E-mail: [398872956@qq.com](mailto:398872956@qq.com)
